# Device entrapment during percutaneous transluminal angioplasty for central venous stenosis in an arteriovenous fistula: a case report

**DOI:** 10.3389/fmed.2026.1807926

**Published:** 2026-06-12

**Authors:** Tongxin Gao, Tao Yang

**Affiliations:** Department of Nephrology, Beijing Zhongguancun Hospital, Beijing, China

**Keywords:** arteriovenous fistula, balloon entrapment, case report, central venous stenosis, percutaneous transluminal angioplasty

## Abstract

Percutaneous transluminal angioplasty (PTA) is a widely used endovascular intervention for arteriovenous fistula (AVF)-associated stenosis. Balloon entrapment is an uncommon but clinically significant complication of endovascular procedures, which may lead to severe adverse events. We report a case of a patient who underwent bare-metal stent implantation for subclavian vein stenosis in 2025 and was re-admitted with limb swelling. Venography confirmed in-stent restenosis of the subclavian vein. During PTA for the target stenotic lesion, structural deformation of the balloon catheter occurred, leading to its entrapment. Multiple attempts at balloon deflation were unsuccessful. Successful retrieval of the damaged balloon catheter was accomplished via percutaneous subclavian vein puncture and percutaneous balloon puncture and decompression.

## Introduction

With the advancement of hemodialysis technology and vascular access creation techniques, the service life of arteriovenous fistulas (AVFs) has been significantly prolonged. However, the incidence of AVF-related complications, particularly central venous stenosis (CVS), has been rising year by year. Percutaneous transluminal angioplasty (PTA) is currently the first-line treatment for CVS, yet it is plagued by low technical success rates and high recurrence rates. In recent years, the development of novel therapeutic techniques and the improvement of medical devices have not only enhanced the success rate of PTA but also enabled the management of more complex vascular lesions. Nevertheless, the risk of procedural complications remains, which is particularly prominent in the treatment of severely stenotic and heavily calcified vessels (1).

Device entrapment is a life-threatening complication defined as a condition in which an implanted device cannot be retrieved from the vascular lumen via conventional maneuvers including advancement and retraction and requires additional instruments or procedural interventions such as a second guidewire, auxiliary balloon, and snare for successful retrieval (2). Device entrapment may lead to vascular perforation, intraluminal thrombogenesis, and in severe cases, massive hemorrhage and pulmonary embolism. Balloon entrapment is an extremely rare complication of PTA that may necessitate emergency surgical intervention, and to date, there is a paucity of evidence-based guidance and clinical recommendations in the published literature. Herein, we report a rare case of balloon entrapment within a previously implanted bare-metal stent placed in the right subclavian vein during PTA for CVS, and describe the emergency management strategy adopted in this case.

## Case presentation

A 60-year-old male patient had a history of type 2 diabetes mellitus and hypertension. Six years ago, the patient underwent autologous arteriovenous fistula (AVF) creation in the right upper extremity for end-stage renal disease due to chronic renal failure. Right upper limb swelling developed 34 months after the AVF surgery. Angiography demonstrated stenosis of the right subclavian vein and right brachiocephalic vein. Percutaneous transluminal angioplasty (PTA) of the right subclavian vein was performed at 35, 41, and 45 months postoperatively, with temporary resolution of limb swelling after each procedure. At 47 months after AVF construction, the patient underwent repeated right subclavian vein PTA combined with implantation of a 14 mm × 80 mm bare-metal stent (Model: WALLSTENT; Brand: Boston Scientific; Manufacturer: Boston Scientific Corporation, MA 01752, United States), and the swelling resolved after the operation.

One month prior to admission, the patient developed recurrent swelling of the right upper extremity and was admitted with a tentative diagnosis of central venous in-stent restenosis. A 7Fr (11 cm) vascular sheath was placed via percutaneous puncture of the basilic vein 2 cm above the right elbow. Venography confirmed severe in-stent stenosis of the right subclavian vein.

A 0.35-inch hydrophilic guidewire (Model: LWSTDA35150; Brand: Merit; Manufacturer: Merit Medical Systems, Inc., West Galway, Ireland) combined with a 4F angiographic catheter was used to cross the stenotic lesion. The guidewire was then exchanged for a 260 cm angled stiff-shaft guidewire (Model: LWSTFA35260EX; Brand: Merit; Manufacturer: Merit Medical Systems, Inc., West Galway, Ireland), with its distal end placed into the inferior vena cava. Over the guidewire via the sheath, a 14 mm × 40 mm balloon catheter (Model: ATLAS, NP 6 atm, RBP 18 atm; Brand: BARD; Manufacturer: Bard Peripheral Vascular, Inc., New York, United States) was delivered.

**Figure 1:**
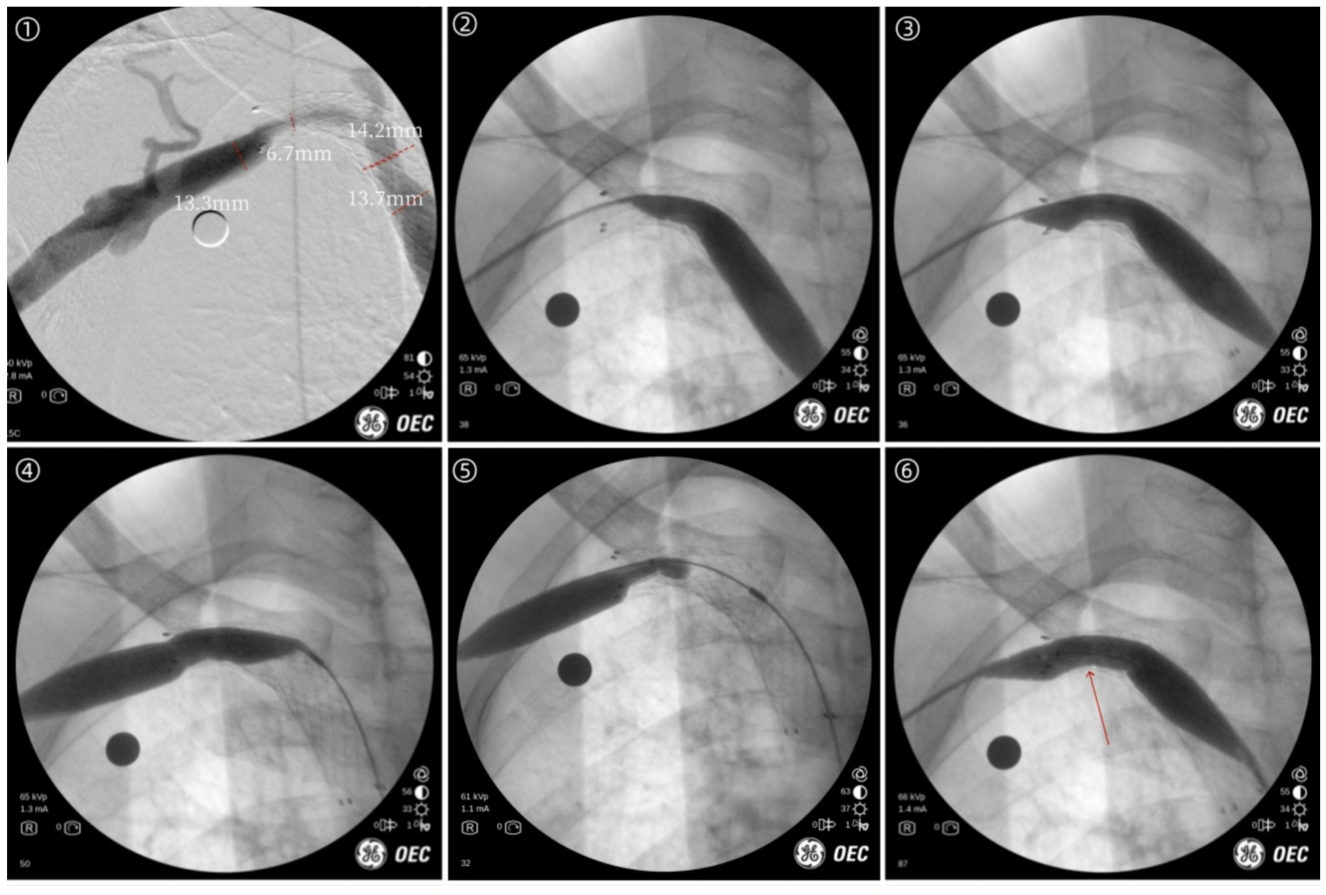
Fluoroscopic images showing the procedural course. **(1)** Venography revealed severe in-stent restenosis of the right subclavian vein; the numbers in the figure indicate the inner diameter values of each blood vessel. **(2–5)** A 14-mm balloon catheter was sequentially inflated for dilatation of the stenotic segment; **(6)** the position indicated by the arrow.

The balloon was positioned at the severely stenotic segment of the right subclavian vein, inflated to the rated burst pressure of 18 atm and maintained for 2 min. Repeated aspiration attempts failed to deflate the balloon. Replacement of the pressure pump still could not achieve balloon deflation. A Y-valve was connected to the balloon deflation port, and multiple deflation devices were applied sequentially, but all attempts ended in failure. Pushing and pulling the balloon catheter were also ineffective, and the catheter remained completely fixed in place (Figure 1).

A V18 guidewire and delivery catheter were inserted again through the sheath, yet repeated attempts failed to pass through the vascular segment at the balloon incarceration site. The stiff tip of the V18 guidewire was used to puncture the balloon inflation lumen via the deflation port for pressure release, which was also unsuccessful.

**Figure 2:**
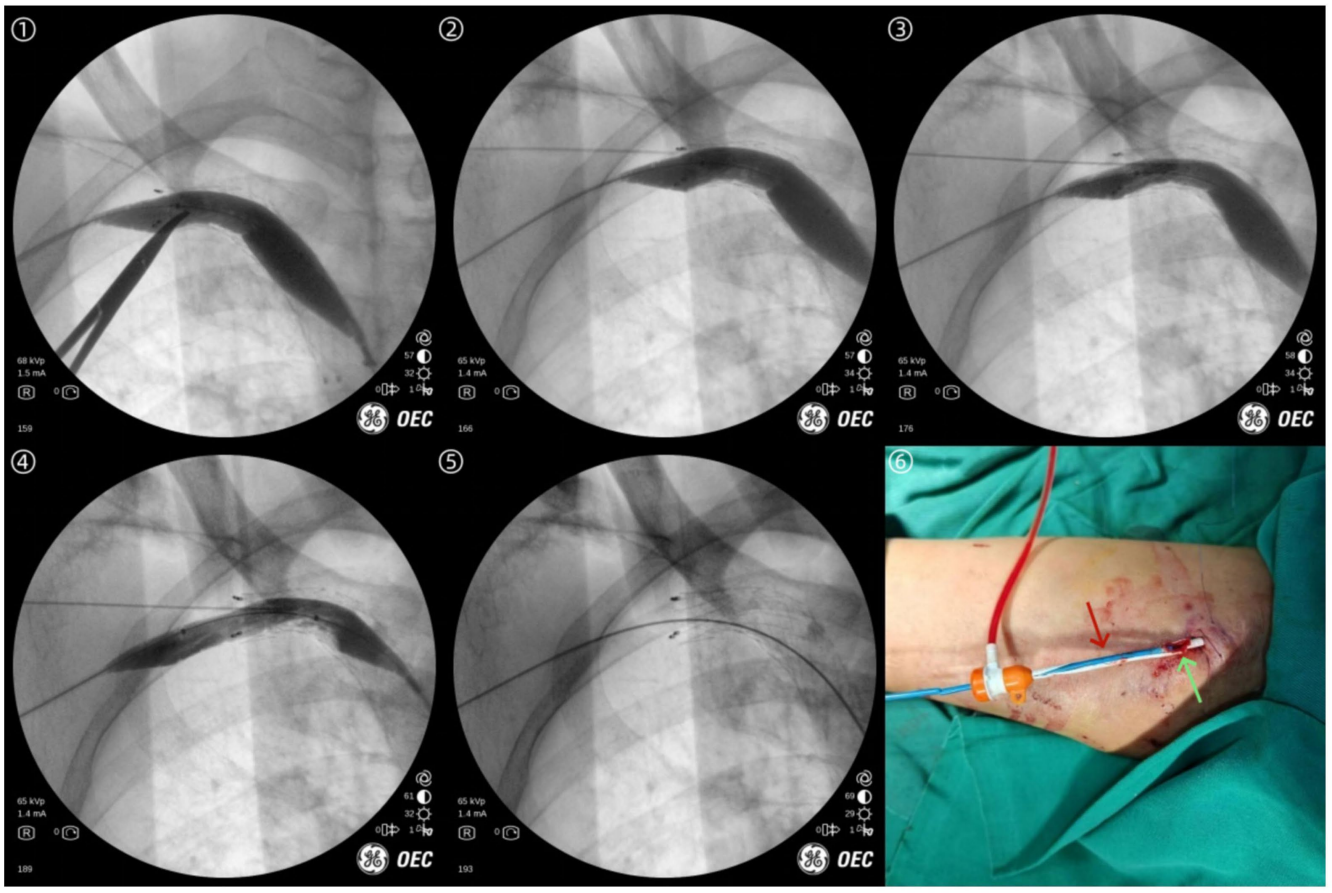
Steps of the balloon catheter retrieval procedure: **(1)** Determination of the percutaneous puncture site was performed with reference to fluoroscopic and anatomical landmarks; **(2–3)** percutaneous puncture was conducted, followed by aspiration of contrast medium from the entrapped balloon to accomplish decompression; **(4)** Adjustment of the puncture site was made, with continued aspiration to ensure complete deflation of the balloon; **(5)** After decompression treatment, the balloon was released from entrapment and withdrew from the subclavian vein. **(6)** The red arrow indicates the externally located segment of the longitudinally incised catheter balloon and catheter sheath being removed simultaneously.

With anatomical localization at the lateral border of the clavicular head of the right sternocleidomastoid muscle, percutaneous puncture of the right subclavian vein was performed using a 22G puncture needle under real-time X-ray guidance. A 10 mL syringe was used to aspirate the contrast agent inside the balloon to relieve intraluminal pressure. Retract the balloon slowly to the vicinity of the basilic vein puncture site, and its delivery shaft was cut off extracorporeally. An 8Fr (11 cm) vascular sheath was exchanged along the guidewire and the intravascular remnant of the balloon shaft. The extracorporeal segment of the sheath was longitudinally incised; the intravascular part of the damaged balloon was traction-fixed at the incision and retracted into the sheath for en bloc removal. Local hemostasis was achieved with purse-string suture (Figure 2).

Postoperatively, the patient’s limb swelling resolved. No local neurovascular injury or other complications were observed, and hemodialysis was continued through the original arteriovenous fistula. During the 1-year follow-up, the patient was rehospitalized at 6 months due to recurrent right limb swelling and received repeat percutaneous transluminal angioplasty (8 mm balloon predilation followed by 14 mm balloon dilation). No recurrent balloon entrapment was noted, and no local neurovascular damage was recorded during the entire follow-up period.

## Discussion

Balloon entrapment may be associated with abnormal vascular anatomical structures, such as vascular tortuosity, angulation, and calcified vascular lesions with sharp edges (3). To date, the exact underlying cause of balloon entrapment remains unclear. Some studies have suggested that potential contributing factors include size mismatch between the balloon catheter and the target vessel, insufficient support force of the guidewire, inadequate structural strength of the balloon, and inadequate pre-dilation (4, 5). Other studies have indicated that, similar to other instruments, forced advancement of the balloon through severely stenotic and calcified lesions increases the risk of balloon entrapment (6). In clinical practice, balloon entrapment mainly occurs in the following scenarios: ① Balloon rupture during PTA, leading to entrapment of the instrument or the damaged balloon catheter at the lesion site; ② Inability of the balloon to deflate due to external force or structural changes of the balloon, resulting in its entrapment at the lesion site (7).

Some studies have proposed that compared with the approach of gradually increasing the balloon size, the initial use of a larger-sized balloon significantly increases the risk of balloon rupture or entrapment (8). As shown in Figure 1, the diameters of the blood vessels adjacent to the stenotic lesion were 13.3 and 13.7 mm, respectively. Instead of using a smaller-diameter balloon for predilation, a balloon catheter with the same diameter as the stent was adopted directly. Combined with the intraoperative manifestations of the patient, we infer that in this case, the large-diameter balloon was compressed by severely hyperplastic vascular intima and the rigid metal framework of the bare stent during inflation. The force-bearing points of the balloon dilation pressure on the vascular stenotic lesion were uneven, and the balloon slipped repeatedly during dilation with difficulty in anchoring the lesion site, resulting in poor dilation efficacy. To anchor the therapeutic segment by traction of the balloon catheter and achieve the expected therapeutic effect, the balloon inflation pressure was increased close to or even up to the rated burst pressure. Under the combined effect of multiple factors, the structure of the balloon was altered, the inflation and deflation port became occluded, the balloon failed to deflate, and balloon incarceration eventually occurred.

Compared with peripheral blood vessels, once central venous balloon entrapment occurs, open surgery may result in significant trauma due to clavicle transection and is associated with multiple complications, and should therefore be used cautiously. For balloon entrapment caused by inability to deflate, some studies have suggested that a Y-type connector can be used to connect two pressure pumps to the balloon to apply negative pressure, increasing the deflation pressure to resolve balloon entrapment (3). However, this method is often ineffective when the inflation port is deformed or even occluded due to balloon structural changes. In addition, some studies have proposed performing controlled rupture of the entrapped balloon. The technique is performed as follows: a second guidewire is placed alongside the entrapped balloon, an over-the-wire balloon is advanced through the guidewire and inflated at low pressure (2–4 atm), the guidewire of the over-the-wire balloon is then removed and replaced with a stiffer guidewire (e.g., Confianza Pro 12 guidewire), and the stiff guidewire is used to puncture the entrapped balloon to deflate it for retrieval (9). This technique requires high operator skill and specialized equipment. It may be difficult to guide a new balloon through the vicinity of the inflated balloon again via a guidewire. There is a high risk of accidental vascular injury during the puncture of the balloon with the stiff guidewire, and it is difficult to puncture non-compliant balloons with the stiff guidewire.

In this study, under real-time X-ray imaging, the contrast medium in the balloon was visualized, allowing real-time marking of the vascular location and precise guidance for the 22G puncture needle to puncture into the subclavian vein and pierce the outer membrane of the entrapped balloon for aspiration and deflation. By observing the puncture needle path in real time, surrounding nerves, pleura, and other tissues were avoided, resulting in a high puncture success rate and significantly reducing the incidence of complications such as hematoma, nerve injury, and pneumothorax. It is an effective measure to treat balloon entrapment resulting from failed contrast medium aspiration. However, longer puncture depth increases the risk of neurovascular injury, especially near the right atrium. Some studies have also proposed using laser ablation to puncture the balloon; however, it should be noted that the heat transmitted by the laser to the balloon may melt the balloon plastic, thereby leading to central venous perforation and more severe medical complications (10).

After the balloon is decompressed via puncture, entrapment can be relieved in most cases. However, once the integrity of the balloon is damaged, it cannot return to its original shape. Blind and forceful traction may injure the central veins and peripheral blood vessels, and even cause the balloon to fracture intravascularly, leading to further safety risks. In addition, the irregular morphology of insufficiently decompressed balloons can increase the risk of vascular laceration near the puncture site. In this study, before withdrawing the balloon, a larger-sized catheter sheath matching the balloon was replaced (8 Fr was mostly selected for the upper limbs, and 9 Fr for the lower limbs). Under real-time X-ray imaging, the balloon was pulled close to the vascular puncture site and retracted into the catheter sheath. The balloon was retracted into the sheath and removed en bloc.

In this case, under real-time X-ray imaging, we performed puncture of the subclavian vein to manage an entrapped balloon caused by failure to aspirate contrast medium. The sheath technique was adopted to retrieve the ruptured balloon without resulting in vascular injury. No long-term complications were observed during postoperative follow-up. It may serve as a rescue option for balloon entrapment in central veins, especially the subclavian vein.

## Data Availability

The original contributions presented in the study are included in the article/supplementary material, further inquiries can be directed to the corresponding author.
